# Dural arteriovenous fistula with spinal dural arteriovenous fistula: a case report and review of the literature 

**DOI:** 10.1186/s13256-023-04170-y

**Published:** 2023-10-24

**Authors:** Peixin Wang, Lele Zhang, Wenqian Zhang, Tiejun Shi, Yikun Sun, Shaojie Cui, Dan Zhang, Fanxuan Kong, Tao Wang

**Affiliations:** 1 Department of Neurosurgery, PLA Strategic Support Force Characteristic Medical Center, Beijing, 100101 China; 2 Department of Spine Surgery, PLA Strategic Support Force Characteristic Medical Center, Beijing, 100101 China; 3 306th Clinical College of PLA, The Fifth Clinical Medical College, Anhui Medical University, Beijing, 100101 China; 4 Core Facility, Center of Biomedical Analysis, Tsinghua University, Beijing, 100091 China

**Keywords:** Dural arteriovenous fistula, Spinal dural arteriovenous fistula, Computed tomographic angiography, Magnetic resonance imaging

## Abstract

**Background:**

This paper analyzed the cases of dural arteriovenous fistula (DAVF) with spinal dural arteriovenous fistula (SDAVF) in the diagnosis and treatment process.

**Case presentation:**

One case involving dural arteriovenous fistula (DAVF) with spinal dural arteriovenous fistula (SDAVF) from the 306th Hospital of PLA was retrospectively analyzed. The patient consulted the doctor due to lower limb sensory and motor disorders while exhibiting symptoms of urinary dysfunction. A computed tomographic angiography (CTA) and cerebral angiography confirmed the diagnosis of dural arteriovenous fistula (DAVF), necessitating surgical treatment. The patient was referred to our hospital for an magnetic resonance imaging (MRI) and a spinal angiography to obtain a confirmed diagnosis for spinal arteriovenous fistula, after which they underwent surgical fistula resection. The invasive intracranial dural arteriovenous fistula (DAVF) resection proceeded smoothly but did not ease the patient's symptoms. However, postoperative symptoms were partially relieved by the lumbar open spinal dural arteriovenous fistula adminstration.

**Conclusions:**

Since not enough is understood about these two diseases, the rate of misdiagnosis is significantly increased. Early diagnosis and treatment of spinal dural arteriovenous fistula (SDAVF) can play a positive role during the recovery from neural function damage.

## Introduction

Dural arteriovenous fistula (DAVF) refers to the abnormal vascular anastomosis between dural arteries and veins, cerebral venous sinus, and cortical veins, which is classified as an intracranial vascular malformation. Spinal dural arteriovenous fistula (SDAVF) is a spinal vascular malformation in which the small arteries that supply the spinal cord or nerve roots communicate directly with the draining veins near the intervertebral foramen on the surface of the spinal cord. These two diseases differ in their location, but both belong to the central nervous system and have similar mechanisms of treatment. Epidemiological investigations have shown [1] that the incidence of SDAVF is (0.5 ~ 1.0) per 100,000, significantly increasing the clinical misdiagnosis and missed diagnosis rates. At present, the clinical DAVF cases combined with SDAVF are rarely reported, and only one case was admitted to our hospital. This was a case of misdiagnosis, leading to the report that follows. Furthermore, its etiology, clinical manifestations, imaging characteristics, and treatment options were analyzed and discussed in combination with the literature to improve the understanding of this type of disease.

## Case presentation

A 57-year-old Han Chinese male patient was admitted to our hospital in July 2017 due to “numbness and weakness in both lower limbs for more than 3 years.” The patient developed bilateral toe numbness without obvious inducement 3 years ago, which gradually expanded to his groin. Then, 2.5 years ago, the patient developed intermittent weakness of the lower limbs, which was induced by climbing stairs or standing for a long time and improved after rest. The symptoms of weakness in both lower limbs developed gradually. The attack cycle became more frequent, and increased to 7–8 times a month. During the attack, no limb pain or convulsions, headaches, dizziness, nausea or vomiting were evident, while he displayed no mental symptoms or disturbance in consciousness. The patient went to the local hospital many times without obtaining a definite diagnosis. Then, 2 years ago, he had trouble defecating (abnormally delayed or infrequent passage of dry hardened feces) once every 2–3 days, and developed difficulty in micturition 6 months ago.

**Fig. 1:**
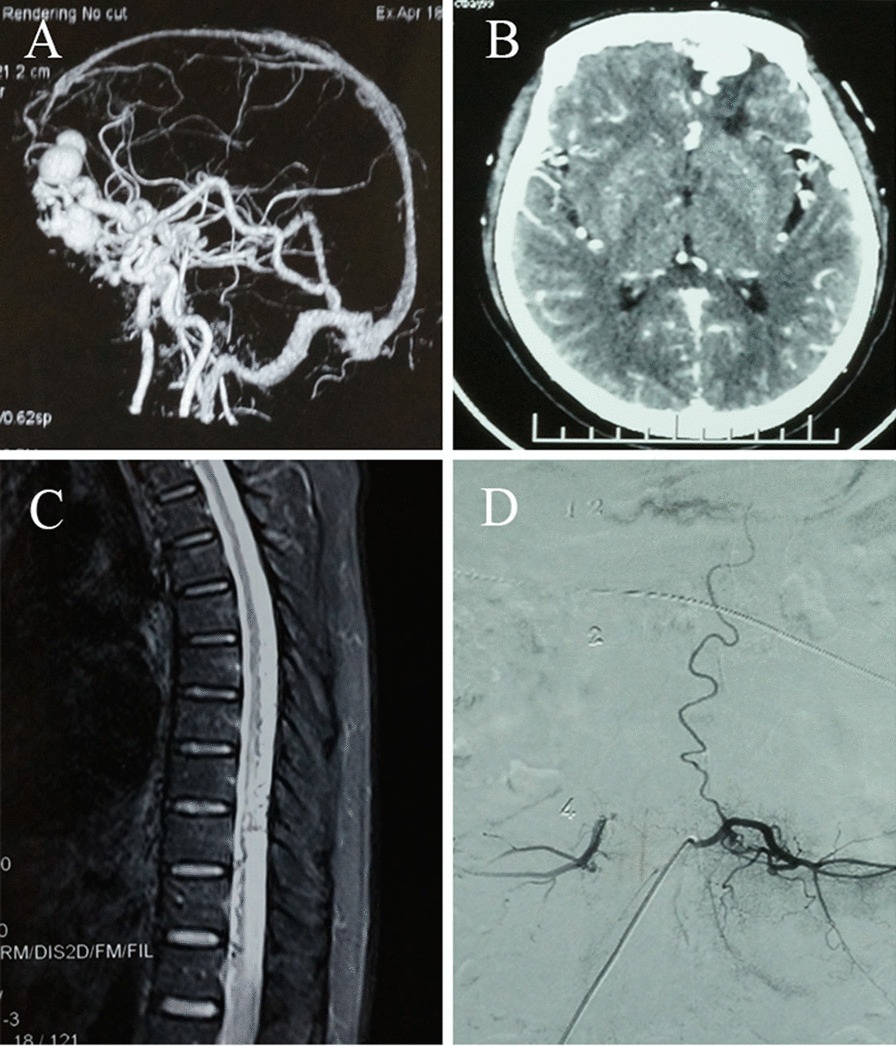
The imaging results of the dural arteriovenous fistula and the spinal dural arteriovenous fistula. **A** Computed tomographic angiography examination of the head shows a space-occupying venous bulb at the frontal base. **B** The axial computed tomographic angiography displays the bottom enhancement of the forehead. **C** Vermiform vascular shadows can be seen in the lower thoracic spinal cord. **D** Digital substraction angiography indicates a thick tortuous upward draining vein

Then, 3 months ago, the weakness worsened in both the patient's lower limbs, and strength in the waist weakened, but he could still walk slowly, experiencing the same numbness as before. Except for limb function, as well as constipation and uroschesis, no additional symptoms of discomfort was apparent. The patient visited a local hospital and underwent a computed tomography (CT) examination of his head to determine the intracranial space occupation, as well as a computed tomographic angiography (CTA) and digital substraction angiography (DSA) examination to identify DAVF (Fig. 1A, B). No special surgical contraindications were found during the examination, and an intracranial DAVF resection was performed.

There was no significant improvement in either the urinary and stool dysfunction or the dyskinesia in both lower limbs after the operation. The patient visited our hospital for further treatment. The physical examination showed that both his upper limbs were V level, while both lower limbs were V_ level. His muscle tone was decreased, the lower limbs were below groin level, the pain and temperature perception had decreased, the anal reflex had weakened, and the bilateral cremasteric emission had disappeared. Furthermore, the bilateral Chadok sign, Babinski sign and Hoffmann sign were negative. Achilles tendon reflex was diminished and ankle clonus test was negative (Table 1). The spinal angiography showed SDAVF (Fig. 1C, D).

**Table1:** Neurological examination result

Neurological examination	
Muscle force	Upper limbs V
	Lower limbs V−
Muscular tension	Decreased
Somatic sensation	Hypoalgesia and thermohypesthesia below the groin level
Anal reflex	Decreased
Cremacteric reflex	Bilateral disappeared
Achilles tendon reflex	Decreased
Ankle clonus test	Bilateral negative
Chadok sign	Bilateral negative
Babinski sign	Bilateral negative
Hoffmann sign	Bilateral negative

After a discussion in the department, an open lumbar SDAVF was performed. During the operation, the fistula was located in the left lumbar third spinal nerves penetrating the spinal dura. The drainage vein extended longitudinally and upward while appearing thicker with a diameter of 2 mm, indicating an arterial change. The fistula of the patient was cut after electrocoagulation. Routine fluid rehydration, volume expansion, and neurotrophic treatment, such as citicoline sodium, were provided after the operation. The muscle strength and muscle tension in the lower limbs of the patient exhibited no significant changes the day after the operation, while numbness increased in the dorsal part of both thighs. Hyperbaric oxygen treatment was performed the second day after the operation. Lumbar muscle spasms subsequently occurred, which could be triggered by lower limbs muscle straining, and hyperbaric oxygen treatment was ceased. The patient was discharged 2 weeks after the operation. However, the numbness in the dorsal part of both thighs showed no signs of improving or progressing, while the symptoms of the lumbar muscle spasms disappeared. During a follow-up consultation 6 months after the operation, the patient still insisted on functional lower extremity exercises. The patient reported a slight recovery from preoperative sensations, but had not returned to normal. The symptoms of the intermittent lower extremity weakness largely disappeared. Constipation and urinary retention were basically absent.

## Discussion and conclusions

### Etiology and pathogenesis

Adult DAVF is an intracranial vascular malformation, which accounts for 10–15% of intracranial vascular malformations. The etiology is not clear and may be related to high-risk factors of thrombosis [2–4], the formation of intracranial venous thrombosis [5–11], tumor compression of the venous sinuses [11], and dural sinusitis [12, 13]. The venous sinus hypertension induces a series of changes that causes the expression of vascular growth factors and promotes the formation of dural neovascularization [6, 13]. SDAVF is a type of spinal vascular malformation and accounts for about 70% of spinal vascular malformations [14]. SDAVF refers to the potential communication branches between the arteries and veins of the dura mater. Under normal circumstances, they are not open. Various factors, such as trauma, can cause the pressure to increase in the blood vessels and veins, opening the potential channels and allowing the arterial blood to pass through the fistula, from where it enters the spinal vein, causing the pressure to rise. In 2001, Kataoka *et al*. [15] reported that the main pathogenic mechanism of SDAVF is venous congestion.

Although the anatomical location of adult DAVF and SDAVF are quite different, etiological analysis indicates that they display a certain similarity and that both represent an acquired disease. In terms of the pathogenic mechanism, both adult DAVF and SDAVF form arteriovenous short circuits. Arterial blood flows into the veins, forming dilated arterialized veins and increasing the venous pressure. In terms of clinical features, the two exhibit apparent differences. Considering the anatomical location of DAVF in the skull, the location and size of the draining vein can have a variety of clinical manifestations. For example, DAVF in the cavernous sinus area can cause exophthalmos and bulbar conjunctival congestion and edema. DAVF that directly drains into the subarachnoid space or cortical vein can cause tumor-like expansion of the vein, which can result in a space-occupying effect and is also one of the causes of spontaneous subarachnoid hemorrhage. Furthermore, sinus drainage from the cortex to the vein, known as downstream, can manifest as fluctuating tinnitus and intracranial vascular murmurs.

The currently recognized pathogenic mechanism of SDAVF is denoted by an arteriovenous short circuit, spinal venous hypertension, and spinal venous blood return disorders. This leads to spinal cord congestion and edema, affecting spinal cord function, and ultimately forming spinal cord avascular necrosis, which results in irreversible neurological dysfunction. Compared with the brain and spinal cord, the low-level nerve center characteristics are mostly manifested as slow onset, progressive aggravation, and ascending limb movement disorders. Consequently, the clinical manifestations of SDAVF are predominantly unitary.

### Diagnosis

During the diagnosis and treatment of this case, the first-diagnosing hospital analyzed the patient's clinical manifestations and prioritized the investigation of craniocerebral diseases. The intracranial lesions were detected via a head CT, after which the intracranial lesions were identified as DAVF using magnetic resonance imaging (MRI) and DSA. So far, the cause of the disease was "cleared," and surgical treatment was performed for DAVF. The operation went smoothly, and the treatment of the intracranial lesions was thorough, but the clinical symptoms did not improve. After the patient arrived at our hospital, the investigation into the spinal cord disease continued, and finally, SDAVF was definitively diagnosed via imaging.

Regardless of the actual clinical work or the data reported in the literature, even simple SDAVF is often challenging to diagnose. Due to its low incidence and no obvious clinical manifestations, it is often misdiagnosed as the spinal-occupying lesion of urinary tract disease, which delays the opportunity for treatment. Alkinson *et al*. [16] reported an average diagnosis time of 23 months for 94 SDAVF cases where many of these patients were misdiagnosed. Two lessons were learned regarding misdiagnosis in the case of our patient. First, the patient exhibited a limb motor sensory disturbance, which extended into segmental aggravation over time, especially the gradual upward shift of the sensory plane, combined with the patient's urinary and stool dysfunction. The location diagnosis should be directed to the spinal cord, and the diagnosis of spinal cord disease should be considered first. Second, the patient's head CT suggested DAVF, requiring surgical treatment. Therefore, the doctor ceased investigation into other causative disease factors. From the perspective of location diagnosis, DAVF cannot fully explain the limb motor sensation disorder and urinary and fecal dysfunction of the patient, necessitating further investigation of the spinal cord disease.

The diagnosis of SDAVF depends on imaging, where MRI reveals a perimedullary vermiform vascular shadow, as well as spinal cord degeneration and edema. Like DAVF, definitive diagnosis depends on the "gold standard," DSA. According to the DSA examination, the fistula of SDAVF is often unique. The arterial blood is directly introduced into the coronary venous plexus of the spinal cord through one or several perforated dural arteries, forming a "short circuit," and finally tortuously expands into the spinal cord surface vein. Therefore, the drainage vein can run the entire length of the spinal cord. Contrarily, DAVF imaging indicated that the blood supply arteries and drainage veins display different degrees of tortuous expansion, since the arterial blood flows directly into the veins and "short-circuits," resulting in excessively high venous sinus pressure and poor cortical venous reflux (CVR). DAVFs that are directly drained by the cortical veins present diffuse cortical vein dilation, tortuosity, and earthworm-like or tumor-like expansion. Draining veins or venous sinuses are often visible during the arterial phase, but the venous sinus circulation time exceeds that of normal circulation time.

### Treatment and prognosis

The surgical treatment of DAVF and SDAVF involve certain similarities, with the closed fistula at their core. For DAVF patients with CVR without intracranial drainage veins, the possibility of serious consequences, such as bleeding, is exceedingly low. Initial observation and conservative treatment measures, such as carotid artery compression are often employed but carry a risk of exacerbating the symptoms. In recent years, with the continuous development of micro-neurosurgery and endovascular interventional treatment techniques, the cure rate of the disease has been significantly increased, while the disability rate has been substantially reduced. Therefore, if there are no specific risk factors, such as old age, severe underlying diseases, difficulty in tolerating surgery, and reluctance to undergo surgery, surgical treatment is recommended. The primary treatment methods involve surgical clamping of the fistula and intravascular embolization.

The main surgical treatment of the two diseases involves craniotomy or hemilaminectomy, clamping or cutting of the fistula. If the anatomical conditions do not allow these techniques, DAVF can also adopt simple external carotid artery ligation, craniotomy, and venous sinus isolation. Surgical treatment is subject to time limitations. DAVF with intracranial hematoma or intracranial venous tumor-like expansion is strong candidates for a craniotomy. Intravascular embolization therapy has gradually become widely accepted and denotes the primary treatment method since it is less traumatic. The aim of interventional therapy is to fill the fistula. DAVF can be treated using the arterial approach, the venous approach, or local puncture embolization of the diseased venous sinus according to the anatomical requirements. The advantage of intravascular embolization is that it is minimally invasive and represents rapid treatment after spinal angiography, but there is a fistula recurrence rate of 15 to 22%. According to reports in the literature, regardless of what treatment is used, as long as the fistula is closed, 90% of patients will experience a rapid improvement from their symptoms, of which dyskinesia and pain are the most significant. Most literature reports [17–19] support the use of interventional vascular embolization for DAVF. If subarachnoid hemorrhage occurs, vascular intervention combined with a craniotomy or simply a craniotomy alone can be selected depending on the condition.

As for the treatment of SDAVF, when anatomical conditions permit, there are no clear indications regarding the choice of surgical treatment. Most of the literature [20, 21] shows that there is no statistical difference in the effective rate of the vertebral closure of the fistula and vascular interventional treatment and that the choice can be made based on the patient's willingness and the maturity of local medical conditions. In this case, although it is confirmed that the lower extremity-related symptoms are caused by SDAVF, this condition should be treated as a priority. However, the intracranial venous tumor has formed, and the related treatment of the DAVF should be administered within a limited time frame. The patient has not fully recovered to a normal state six months after the operation. The reason may be the irreversible damage of spinal cord function caused by the long-term ischemic edema of the spinal cord, which is similar to the more severe cases of chronic progressive neurological dysfunction reported in most literature. Due to the low incidence of SDAVF, the current research focuses on early diagnosis and treatment methods, and few studies are available involving postoperative physical rehabilitation treatment. However, similar to the rehabilitation methods used for slowly progressive neurological disorders, we think personalized physical rehabilitation therapy is beneficial to the recovery of limb and urinary dysfunction after SDAVF.

Regardless of the treatment method, if the nerve function is irreversibly damaged, the postoperative effect will be poor. In this case, the patient's neurological dysfunction only partially recovered after treatment. The main reason was that the clinical symptoms were mistakenly attributed to DAVF during the initial diagnosis. In fact, the patient's neurological damage was attributed to the SDAVF. Many studies [22, 23] pointed out that DAVF and SDAVF should be diagnosed and treated as early as possible by blocking the blood flow to the fistula, reducing the pressure in the cortex, and intramedullary vein, alleviating edema and ischemic changes and reducing clinical symptoms and nerve function damage. Regarding clinical characteristics, the patient has progressive, lower limb sensory and motor disorders, as well as signs of urinary dysfunction, while the location diagnosis points to the spinal cord. Although the detection of craniocerebral lesions has been prioritized, imaging investigation of the spinal cord should not be abandoned based on the localized diagnosis. The patient's DAVF was also more severe, requiring surgery. Therefore, the ideal treatment plan for this case is to prioritize the SADVF, while the second stage should deal with the DAVF. In short, the exploration of the optimal treatment methods for DAVF and SDAVF and the study of factors affecting the prognosis of patients require continued observation and follow-up measures in multiple treatment centers.

## Data Availability

The author will supply the relevant data in response to reasonable requests.

## Abbreviations

DAVF: Dural arteriovenous fistula
SDAVF: Spinal dural arteriovenous fistula
CVR: Cortical venous reflux
CTA: Computed tomographic angiography
DSA: Digital substraction angiography
CT: Computed tomography
MRI: Magnetic resonance imaging
